# Primary Circumscribed Meningeal Melanoma Involving the Meckel’s Cave: A Report of a Rare Case and Review of Literature

**DOI:** 10.7759/cureus.55427

**Published:** 2024-03-03

**Authors:** Syeda Sara Tajammul, Salim Chaib Rassou, Javeria Munir, Zubair Ahmed, Asma Naaz Nadaf

**Affiliations:** 1 Radiation Oncology, Sultan Qaboos Comprehensive Cancer Care and Research Centre (SQCCCRC), Muscat, OMN; 2 Radiology, Sultan Qaboos Comprehensive Cancer Care and Research Centre (SQCCCRC), Muscat, OMN; 3 Pathology, Sultan Qaboos Comprehensive Cancer Care and Research Centre (SQCCCRC), Muscat, OMN

**Keywords:** trigeminal, meckel’s cave, circumscribed, pigmented, primary meningeal melanocytic tumors, melanoma

## Abstract

Primary intracranial meningeal melanomas are rare. Diagnosing primary meningeal melanomas mostly involves comprehensive assessment through clinical and radiological means. This evaluation should encompass a detailed dermal and ophthalmic examination. Any suspicious lesion must be biopsied and examined microscopically. This is crucial not only to differentiate primary intracranial melanoma from other brain tumors but also to rule out metastases from potential sources of primary cutaneous or non-cutaneous melanomas. Surgery is considered the mainstay of treatment. Despite melanomas being generally considered radio- and chemo-resistant tumors, adjuvant radiotherapy and chemotherapy still play a crucial role in their management. The treatment landscape for primary meningeal melanoma is continually evolving, with ongoing research aiming to improve outcomes for patients with this challenging disease.

## Introduction

Primary intracranial meningeal melanomas are rare entities, compromising 1% of all melanoma cases and a mere 0.07% of all brain tumors [1]. Primary malignant meningeal melanoma of the Meckel’s cave is even rarer [2]. The prognosis of primary malignant meningeal melanoma can be much better if complete resection is achieved as compared to the patients with CNS metastases from melanomas of other sites [3]. Although these tumors are considered chemo and radioresistant, adjuvant radiotherapy and chemotherapy play a critical role in their management [4].

We report a case of primary malignant melanoma of the Meckel's cave in a 31-year-old lady which was diagnosed by histological examination of the excised mass. We discuss the characteristics of a Meckel's cave melanoma on magnetic resonance images and histopathology. Literature is specifically reviewed about the management of the disease.

## Case presentation

**Figure 1:**
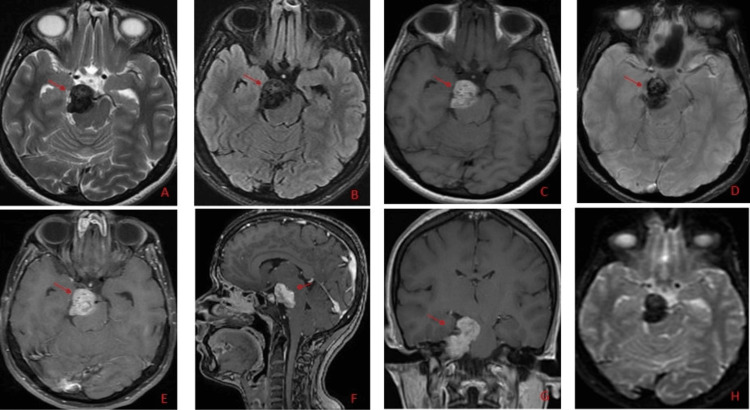
Pre-operative MRI brain showed a well-defined extra-axial lobulated mass (red arrows) measuring 3.7 × 3.6 × 2.1 cm mass in the right trigeminal cisterna (T2 axial image - A, FLAIR axial image - B), post-contrast T1 fat sat images - (sagittal image F and coronal image G) with extension into Meckel’s cave. The tumor encased the right posterior cerebral artery causing a mass effect on the adjacent structures (image E). There was no extension in the internal auditory canal. There was no diffusion restriction (H). It appeared hyperintense on T1-weighted images (C) and hypointense on GRE images (D), suggestive of a bleed FLAIR: Fluid-attenuated inversion recovery

A 31-year-old lady presented to a private hospital with a one-year history of headache not responding to analgesics. It was not associated with any other symptoms. Pre-operative MRI brain showed a well-defined extra-axial lobulated mass (red arrows) measuring 3.7 × 3.6 × 2.1 cm in the right trigeminal cistern (Figure 1).

**Figure 2:**
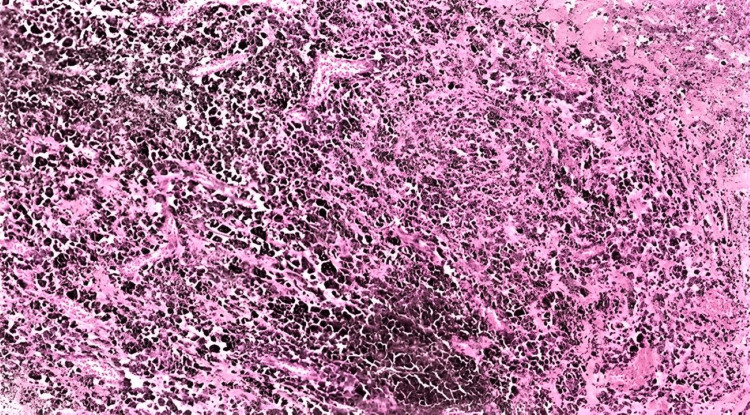
H&E (x100, low power), heavily pigmented neoplasm with cytological features obscured by the melanin pigment

**Figure 3:**
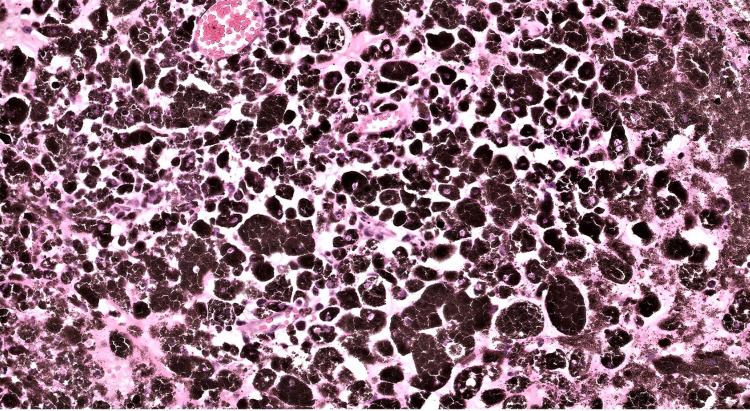
H&E (x200), tumor cells exhibiting large irregular variable vesicular and hyperchromatic nuclei

She underwent right retro-mastoid craniotomy and excision of the lesion. Microscopic examination showed a heavily pigmented neoplasm. The morphology was obscured by the heavy pigmentation. The less pigmented areas showed epithelioid cells arranged in sheets and vague lobules in a focally fibro-collagenous background (Figure 2). Neoplastic cells showed nuclear pleomorphism with prominent eosinophilic nucleoli (Figure 3). Immunohistochemistry (IHC) markers like S100, SOX10, HMB 45, and Melan A along with KI-67 were attempted but were non-contributary due to heavy pigmentation. Based on histological features and the location of the neoplasm, the differential diagnosis included melanocytoma of intermediate grade, primary meningeal malignant melanoma, and malignant melanotic nerve sheath tumor.

She was referred to Sultan Qaboos Comprehensive Cancer Care and Research Centre (SQCCCRC) for further investigations and treatment. At SQCCCRC a detailed dermatological and clinical examination of the entire body revealed no other lesion. Her detailed ophthalmological examination performed to rule out the possibility of primary uveal melanoma, was also unremarkable. CT scan of the orbits was also negative.

**Figure 4:**
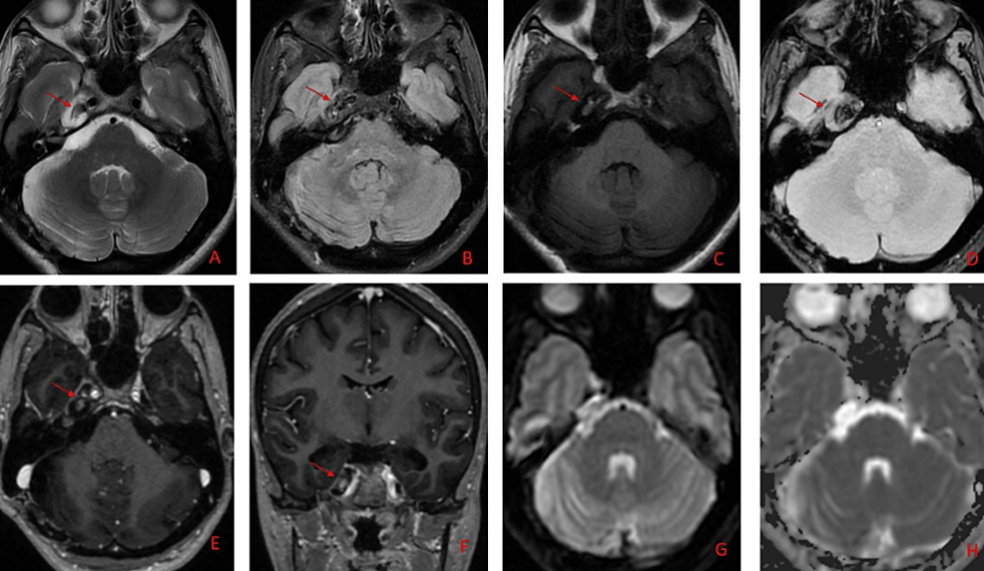
Post-operative MRI brain showed a residual lesion in the right Meckel's cave having hypointense signals to gray matter on T2 (image A) and FLAIR (image B), gradient imaging (D), and hyperintense signals on T1 WI (C). It abuts the right seventh and eighth nerve complex and a cavernous segment of the internal carotid artery. Minimal enhancement on axial (E) and coronal (F) post-I/V Gadolinium contrast images was also observed. No restriction on DWI (G) and ADC (H) images DWI: Diffusion-weighted imaging; ADC: Apparent diffusion coefficient; FLAIR: Fluid-attenuated inversion recovery; WI: Weighted imaging

**Figure 5:**
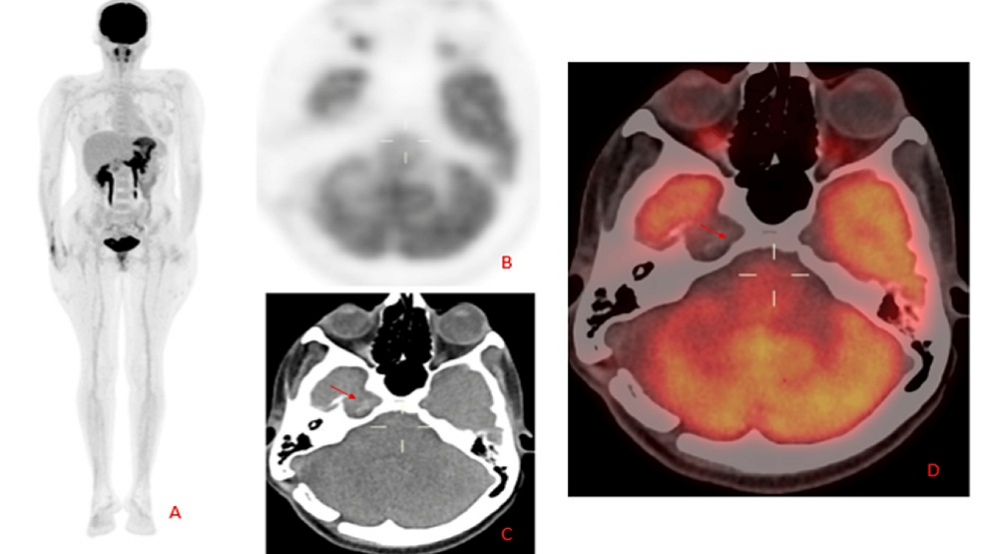
PET scan showing no evidence of distant metastasis (image A). Hyperdense area on PET CT image B and (red arrow in) image C within the right Meckel's with no significant increase FDG uptake (red arrow in image D) FDG: Fluorodeoxyglucose

She underwent a post-operative MRI brain which reported a residual lobulated lesion (Figure 4). A PET scan was done to rule out any other primary site of origin that showed no evidence of any abnormal hypermetabolic visceral or bony lesion (Figure 5).

The case was sent abroad for review and the final histopathology report received described a heavily pigmented neoplasm in which the cytological details in many areas were obscured. In less pigmented areas tumor cells appeared to have large, irregular variably vesicular, hyperchromatic atypical nuclei. The characteristic nuclear groves of melanotic Schwannian cells were not seen. IHC showed focal positivity for S100 along with multifocal positivity for SOX10, Melan A, and HMB45 while SMA and desmin were negative. Staining for PRKARA was positive and that for BAP-1 was equivocal. The final impression was that of a malignant melanoma.

**Figure 6:**
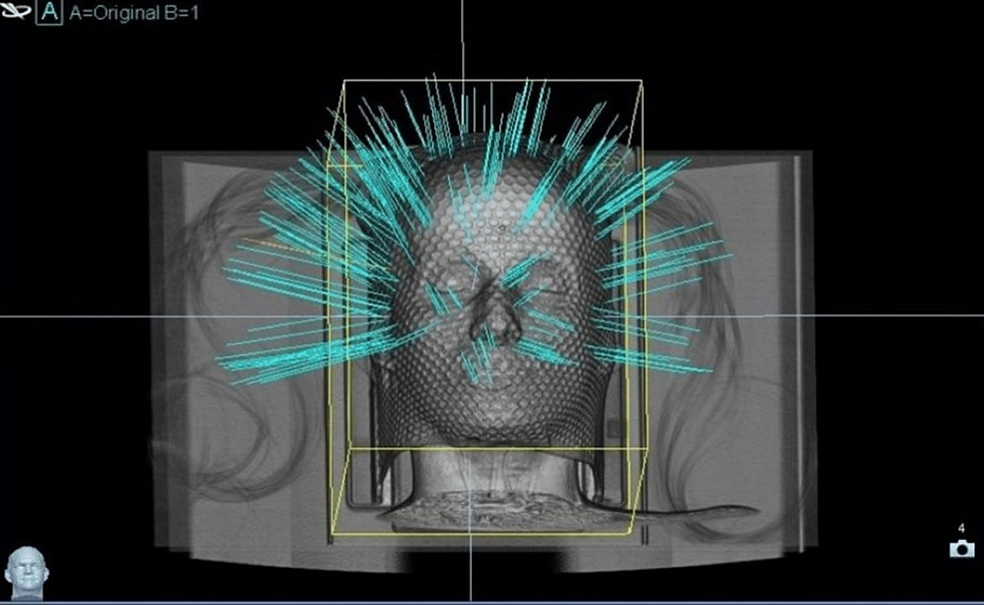
Cyberknife treatment set up showing head position with a thermoplastic sheet. Cyan lines represent the beam entrance points

**Figure 7:**
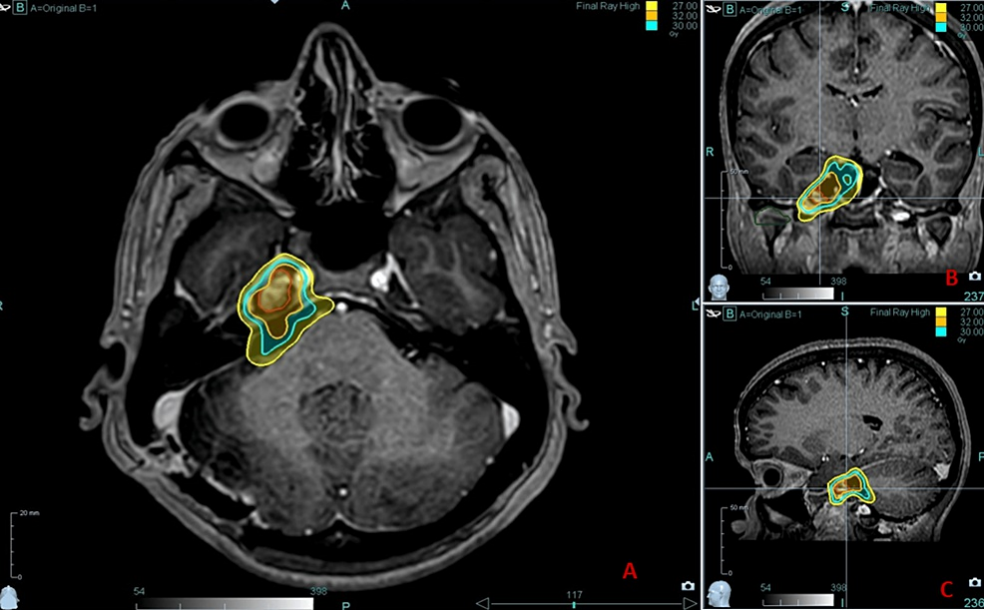
Radiotherapy treatment planning CT images showing the target with isodose lines in (A) axial image (B) coronal image, and (C) sagittal images

Neurosurgery consultation was taken for the re-excision of the residual disease in Meckel’s cave. The lesion was deemed inoperable, and the patient was advised for radiotherapy. She was treated with stereotactic radiotherapy by cyber knife with a dose of 30 Gy in five fractions (Figures 6, 7). Molecular profiling of the tumor was performed by CARIS including BRAF, however, no specific genetic alteration was detected. Due to the presence of residual disease, the patient was started on post-operative Pembrolizumab.

**Figure 8:**
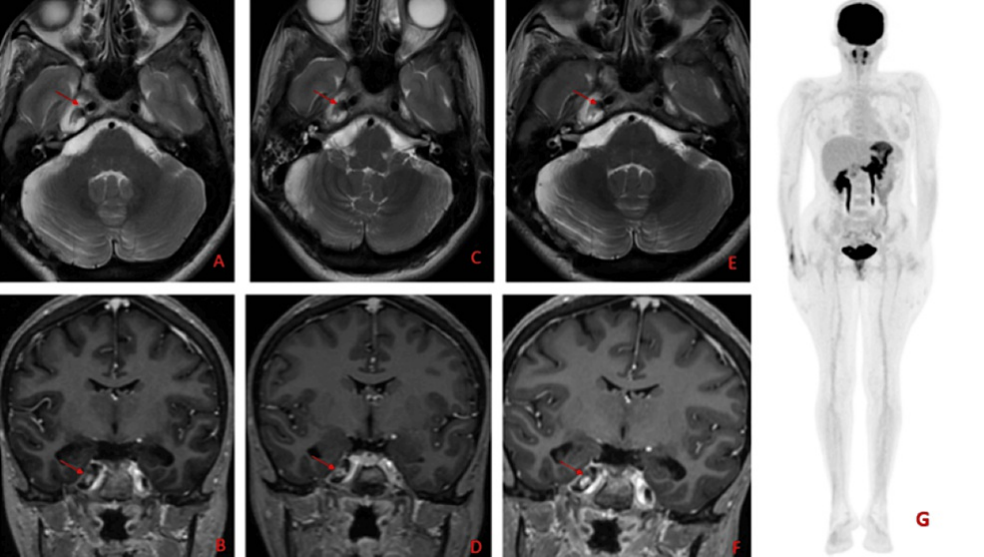
Serial MRI brain done on follow-ups at three months (images A and B) six months (images C and D) nine months (images E and F) showing stable disease marked by red arrows and PET scan at 12 months showing no evidence of distant metastasis (image G)

Her serial MRI brain done at three monthly intervals showed stable disease with no significant interval change in appearance, size, or signal characteristics of the lesion. A PET scan after the completion of one year also showed no evidence of distant metastasis (Figure 8).

## Discussion

Melanoma results from the malignant transformation of melanocytes which are derived from neural crest cells. During the initial phases of development, these neural crest cells can migrate to different locations which are potential sites for the development of melanocytic lesions in the future [5]. Cutaneous melanomas are significantly more common than non-cutaneous melanomas which typically originate from ocular, mucosal, and other infrequent locations or rare sites [6]. Primary intracranial meningeal melanomas are one of the rare entities [1]. Bhandari et al. attributed the first case of primary solitary intracranial melanoma to Oogle in 1899 [7]. Melanomas of Meckel’s cave are even rarer [2].

According to the 2021 WHO classification of CNS tumors, primary intracranial meningeal melanoma is broadly categorized as diffuse meningeal melanocytic neoplasms and circumscribed meningeal melanocytic neoplasms. This latter category includes benign meningeal melanocytoma and its distinctly malignant form, meningeal melanoma [8]. In the brain, melanocytes are primarily located in the pia mater surrounding the medulla and upper cervical cord, but they can also be found as heterotopic masses of melanocytes within the brain parenchyma [5]. The Meckel's cave and cranial base have a peculiar predilection for primary meningeal melanocytic neoplasms, as in our case [9].

Meningeal melanomas typically develop during the fourth and fifth decades of life, are more frequent in males, and, like other melanomas, are more prevalent among whites and less common among black and Asian populations [5,7]. Our patient showed a slight deviation from these statistics, as she is 30 years old, a female, and of Asian origin. Depending on their origin, primary meningeal melanomas can either develop de-novo or within the context of neurocutaneous melanosis [10]. Regarding our patient, she denied any personal or family history of melanoma as well as any history of excessive sun exposure. 

Patients with circumscribed meningeal melanocytic lesions generally present with focal neurological signs and meningism [7]. Patients involving Meckel’s cave usually present with trigeminal neuralgia. However, the patient might present with other symptoms without compromising the trigeminal nerve [11]. In our case, the patient initially presented with a headache without any features of trigeminal neuralgia.

Due to their rarity, distinguishing between primary and metastatic melanomas based solely on histological and neuroimaging features is often very difficult. In his important paper published in 1976, Hayward established criteria for categorizing tumors into primary melanomas, secondary tumors, and melanin-containing variations of other intracranial tumors. According to Hayward's criteria, a solitary intracranial meningeal melanoma is more likely to be primary, but on the condition that no melanocytic lesion is identified outside the central nervous system (CNS) [12]. It is important to keep in mind, though, that the well-known but rare phenomenon of spontaneous regression of cutaneous melanoma in the presence of a space-occupying melanocytic lesion in the brain may lead to an erroneous diagnosis of the primary and should be considered in the differentials while looking for the primary meningeal melanoma [13].

In our case, a detailed dermatological, ophthalmological, gynecological, histopathological, and radiological evaluation of the patient was done to rule out neurocutaneous melanosis and to exclude any primary site in the skin, eyes, or mucosa. However, no abnormality was detected elsewhere. Based on this, a diagnosis of primary circumscribed meningeal melanoma was rendered.

Histologically melanomas are usually highly cellular, more pleomorphic, and mitotically active, and show areas of coagulative necrosis. They are composed of spindled epithelioid cells with variable cytoplasmic melanin and demonstrate unequivocal invasion of adjacent brain parenchyma. On IHC, Melan A, HMB 45, S100, vimentin, and SOX 10 are all diffuse strong positive. Differential diagnosis of primary meningeal melanomas on histological examination includes melanocytoma of intermediate grade and malignant melanocytic nerve sheath tumor (previously called melanotic schwannoma) [14].

Among the various imaging techniques, contrast-enhanced magnetic resonance imaging (MRI) is preferred over a CT scan as it is more sensitive. On MRI, melanomas typically exhibit a classic hyperintense appearance on T1-weighted and hypointense T2-weighted images. This appearance is due to the presence of melanin which contains paramagnetic elements such as copper, manganese, and zinc causing T1 signal shortening. Around 50% of melanomas tend to experience hemorrhage, contributing further to T1 signal shortening, particularly based on the melanoma's age [15]. Fluorodeoxyglucose positron emission tomography (FDG PET) is significant in helping to exclude disease outside the CNS. Whole-body FDG PET demonstrates approximately 91% specificity in detecting locoregional disease and in staging melanomas [16].

Surgery plays a major role in the management of primary meningeal melanomas, as it does for other CNS tumors. The goal of surgery is a maximally safe resection, which has a significant impact on survival outcomes for patients with these tumors [3]. Although melanomas are generally considered radio- and chemo-resistant tumors, adjuvant radiotherapy and chemotherapy still play a crucial role in their management. A study by Arai et al. clearly showed that patients who underwent gross tumor resection (GTR) along with adjuvant radiotherapy and/or chemotherapy had significantly higher one-year and five-year overall survival rates (73.0% and 40.1%, respectively) and a longer estimated median survival time of 53 months compared to patients who had GTR alone (20.5 months) or received RT and/or chemotherapy without GTR (13.0 months) [4]. Melanoma cells are more responsive to higher doses per fraction (hypofractionation) of radiation. This concept is supported by the low value of the α/β ratio (2.5 Gy) in the linear-quadratic model [17]. Based on this concept, a stereotactic hypo-fractionated regimen of 30 Gy in five fractions was given to our patient.

Melanoma's inherent chemoresistance, coupled with the difficulty of drug penetration through the blood-brain barrier and the expression of active efflux transporters such as P-glycoprotein and breast cancer resistance protein, creates hurdles for effective chemotherapy in CNS melanomas [18]. Various chemotherapeutic agents like dacarbazine, interferon, temozolamide, etc. have been explored with mixed outcomes [3,19]. Targeted therapies like kinase inhibitors for BRAF and MEK, as well as immune-checkpoint inhibitors, have shown promise in melanoma brain metastases and hold promise for extrapolation in primary meningeal melanomas as well. Based on this our patient is started on pembrolizumab [19].

In a retrospective cohort of 24 patients Byun et al. concluded that regardless of the therapeutic modalities used, the overall survival rates of the primary intracranial melanomas were 100% at 6 months and dropped to 25% at 18 months of follow-up. Similarly, at three months, the progression-free survival rate was 66.7%, and at 12 months, it had dropped to 16.7% [20].

The treatment landscape for melanoma is continually evolving, with ongoing research aiming to improve outcomes for patients with this challenging disease.

## Conclusions

Diagnosing primary meningeal melanoma is indeed challenging and requires a high index of clinical suspicion along with detailed radiological and histopathological evaluation. Due to their rarity, there are currently no specific guidelines or published randomized trials for their management. In this regard, the role of a multidisciplinary team of medical professionals experienced in oncology is extremely important to make the best possible decision based on available experience and individual patient considerations.
